# Effectiveness of depression and anxiety prevention in adolescents with high familial risk: study protocol for a randomized controlled trial

**DOI:** 10.1186/1471-244X-13-316

**Published:** 2013-11-22

**Authors:** Sanne PA Rasing, Daan HM Creemers, Jan MAM Janssens, Ron HJ Scholte

**Affiliations:** 1 Behavioural Science Institute, Radboud University Nijmegen, P.O. Box 9104, 6500 HE Nijmegen, Netherlands; 2 GGZ Oost Brabant, P.O. Box 3, 5427 ZG Boekel, Netherlands

**Keywords:** Prevention, Indicated, Selective, Depression, Anxiety, Adolescents, High risk

## Abstract

**Background:**

Depression and anxiety disorders during adolescence can have detrimental consequences. Both disorders are related to negative outcome in various areas during adolescence and are also predictive of depression and anxiety disorders later in life. Especially parental psychopathology and being female are risk factors that increase the probability of developing one of these disorders during adolescence. Research has shown that prevention programs have promising results, especially for adolescents who have these risk factors. Therefore, in this study, we will focus on the effectiveness of a prevention program ‘A jump forward’ that has been developed for adolescent girls with a familial risk of depression and/or anxiety.

**Methods/Design:**

We designed a randomized controlled trial to test the effectiveness of an indicated and selective prevention program aimed at depression and anxiety in adolescent girls. Adolescents aged between 11 and 15 years old with depressive and/or anxiety symptoms and with parents who show indicators of parental psychopathology will be randomly assigned to the experimental (N = 80) or control groups (N = 80). Participants in the experimental group will follow a preventive intervention, consisting of six sessions of 90 minutes each. All participants will complete baseline, intervention phase 1 (after session 2), intervention phase 2 (after session 4), post-intervention, 6 month follow-up, and 12 month follow-up assessments. Furthermore, parents will be asked to complete assessments at baseline, post-intervention, and 12-month follow-up. Primary outcome will be depressive symptoms. Secondary outcomes will be anxiety symptoms, suicidal ideation, response style, negative cognitive errors, parental emotional support and parental control, parental psychopathology, parenting stress and adolescents’ depression and anxiety symptoms according to the parents.

**Discussion:**

This paper described the study designed to evaluate a program for preventing depression and/or anxiety in high-risk adolescents over a 12-month follow-up period. If the program showed to be effective in reducing symptoms of depression and anxiety and preventing adolescents from developing clinical levels of these disorders, our results would be relevant to practice. Thus, the intervention could be used on a large scale. Moreover, this study aims to contribute to the evidence-based prevention of depression and anxiety of adolescents.

**Trial registration:**

Dutch Trial Register NTR3720

## Background

Depression and anxiety disorders are a major public health concern. The prevalence of clinical depression in adolescents is approximately 5.6% [1]. Regarding anxiety disorders, prevalence rates in children and adolescents vary from 3% to 20% [2]. In addition, lifetime prevalence of depression in adolescents is suggested to be 28.8% and of anxiety disorders, lifetime prevalence is estimated at 16.6% [3]. These numbers do not even include children and adolescents with increased levels of depressive and anxiety symptoms, which are just below the diagnostic threshold [3].

Depression and anxiety disorders during adolescence can have detrimental consequences. That is, both disorders at this age are related to negative outcomes in various areas, such as poor psychosocial functioning [4], impairment in social relations [5], poor academic performance [4,5], and an increased risk for substance abuse [4]. Furthermore, adolescent depression and anxiety are both associated with depression, anxiety disorders, and even suicide later in life [6,7]. Not only clinical levels of depression or anxiety have negative outcomes, also subclinical levels of depression or anxiety are associated with significant distress and dysfunction, and they are also a risk for future disorders [7-9].

It is important to examine the role of risk factors to understand the onset and maintenance of psychopathology during adolescence. With respect to depression and anxiety disorders, several factors increase the probability of developing one of these disorders. The first important risk factor is parental psychopathology, as children of parents with a depression are three times more likely to develop an episode of a major depressive disorder compared to children whose parents do not have this disorder [4,10,11]. With regard to anxiety, 68% of children of parents with this disorder show symptoms of an actual anxiety disorder [12]. Furthermore, these children are two to seven times more likely to develop an anxiety disorder compared to children of parents without an anxiety disorder [13-17]. Moreover, children of parents with an anxiety disorder, a depression, or both, have significantly higher odds of developing depression or anxiety compared to children of parents without these disorders [16]. The first explanation is that these children experience more stress at home and that their parents have less parenting skills. Nevertheless, the relations between particular patterns of depression and anxiety symptoms in adolescents on the one hand and parental psychopathology, parenting characteristics, parental emotional support, and parental control on the other hand remain to be further specified. The second explanation is that a genetic predisposition increases the probability of developing mood and anxiety disorders. Theorists assume that depression and anxiety disorders are familial disorders and partly the result of a genetic heredity. However, the largest part of evidence suggests that depressive and anxiety symptoms rather than specific disorders are heritable. This genetic influence accounts for 30-50% of the variance in these symptoms [18-23]. The second risk factor in the development of depression and anxiety during adolescence is gender. Females have been identified as being at a higher risk of developing depression and anxiety disorders [24-26]. Differences between boys and girls seem to arise during puberty, and particularly adolescent girls are vulnerable to develop a clinical depression [27] and often show subclinical depressive and anxiety symptoms [28].

Given the high prevalence rates of depression and anxiety disorders in children and adolescents as well as the effect these disorders have on their current and future psychosocial functioning, many treatment programs have been developed. Most of these programs are based on cognitive behavioral therapy (CBT), which addresses, for example, dysfunctional emotions, maladaptive behaviors, and cognitive processes. That is, these negative cognitions generally result in higher levels of depression and anxiety. This implies that response style and cognitive errors are assumed to mediate the relation between prevention programs and symptoms of depression and anxiety. CBT is known to be effective in treating and preventing depression and anxiety in adolescents [29-31] individually as well as in groups [32,33]. Importantly, it has been shown that the coping skills learned from a CBT intervention could mediate the relation between family stressors and the actual development of adolescent depression or anxiety disorders [34].

Adolescents with symptoms of depression and/or anxiety who have parents with elevated levels of depression and/or anxiety have a relative high risk to develop depression or anxiety disorders later in life. Because children of parents with a mental illness often develop maladaptive self-schemas as a consequence of negative family-related events, CBT would be a preferred intervention [34]. Despite these facts, to date, only a limited number of studies have been conducted to examine the prevention of depression and/or anxiety disorders in adolescent offspring of depressed or anxious parents by means of CBT [35-38]. The findings showed that preventive interventions had positive outcomes, such as decreased symptomatology. However, none of these preventive interventions targeted both depression and anxiety, although these disorders have a high comorbidity, simultaneous development [39,40], and a considerable overlap in symptoms, especially during adolescence [41]. Since the aetiology and pathogenesis of depression and anxiety also overlap substantially, we argue that the prevention programs should focus on both disorders simultaneously, specifically during adolescence. Therefore, common factors can be prevented, and there might be greater potential to obtain benefits [42,43].

The current study combines indicated prevention and selective prevention [44], which respectively refer to the prevention provided to adolescents with elevated levels of depression or anxiety and to the prevention provided to targeted subgroups distinguished by specific traits, in our case children who have parents with elevated levels of depression or anxiety. As mentioned above, these adolescents have an extra high risk to develop a depression or anxiety disorder. The adolescents allocated to the experimental group will undergo a preventive intervention that consists of six weekly meetings of 90 minutes each. It will focus on acquiring knowledge of depression and anxiety, learning to recognize and distinguish emotions, dealing with difficult situations, solving social problems, and creating and using a supportive social network. Throughout the program, the adolescents will complete various exercises based on cognitive behavioral therapy, behavioral activation, and exposure.

The primary aim of this study is to test the effectiveness of the program ‘Een Sprong Vooruit’ [A Jump Forward] in preventing depressive and anxiety symptoms among adolescents with elevated symptoms of depression and anxiety who have parents with elevated symptoms of depression and/or anxiety. The secondary aim is to determine whether response style and cognitive errors mediate and whether parental psychopathology, parenting stress, parental emotional support and parental control moderate the effectiveness of the prevention program.

## Methods

The study design will be reported in accordance with the CONSORT 2010 statement for reporting parallel group randomized trials [45]. The medical ethics committee CMO Region Arnhem-Nijmegen, The Netherlands, has approved this study.

### Design

The present study is designed as a non-blinded randomized controlled trial (RCT) with two conditions (intervention versus control). Adolescents will be screened for depression, anxiety, suicide ideation, and parental psychopathology in order to select high-risk adolescents. The program is designed for indicated and selective prevention [44]; therefore, adolescents with elevated symptoms of depression and/or anxiety as well as with a parent who has elevated levels of depression or anxiety symptoms will be selected and recruited.

**Figure 1:**
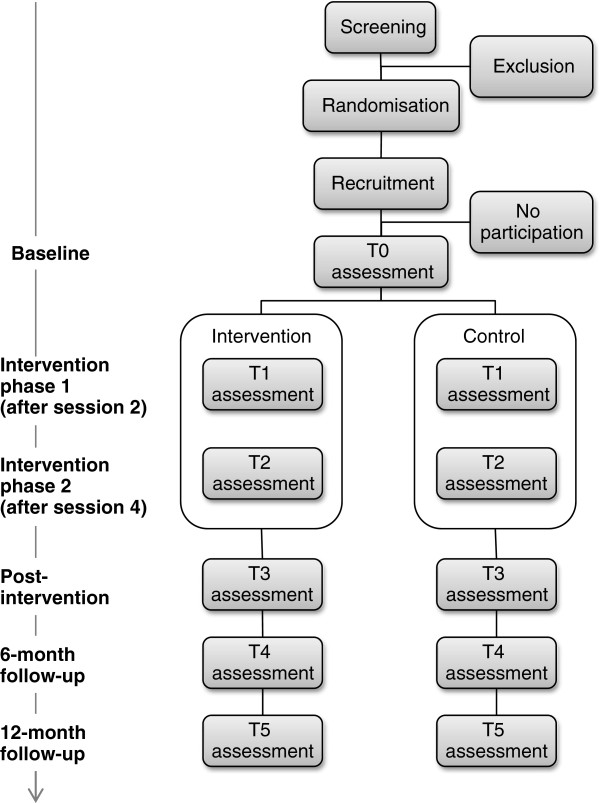
Overall study design.

After the completion of screening and recruitment, participants will be randomized to either intervention or control group. The assessments will be conducted at baseline (T0), during the intervention phase after session 2 (T1), during the intervention phase after session 4 (T2), at post-intervention (T3), at 6-month months follow-up (T4), and at 12-month follow-up (T5). The overall study design is captured in Figure 1.

### Participants’ eligibility

Adolescents with depressive and/or anxiety symptoms who are willing to participate in a prevention program will be eligible for this study. Inclusion criteria are age between 11-15 years old; increased levels of depression and/or anxiety; at least one of the parents showing symptoms of a depression or anxiety disorder; and both adolescent and parents having sufficient knowledge of the Dutch language. Exclusion criteria are the absence of parental permission; adolescent already receiving treatment for mental health problems; and presence of prominent suicidal ideation (score 2 on CDI item: a desire to kill oneself, if given the chance).

### Recruitment

Students in their first and second year of secondary school, from vocational training up to pre-university level, will receive written information about the screening and the study. After receiving passive consent, these students will be screened for depression, anxiety, and parental psychopathology. Students who meet the inclusion criteria will be randomly assigned to one of the two conditions. After that, the adolescents and their parents will receive verbal and written information about the study and written informed consent from adolescents and parents will be obtained.

### Sample size

The sample size is based on the expected difference (Cohen’s d = 0.50) on the primary outcome variable between the experimental and control group at 12 months follow-up (based on a meta-analytic review [46]). With an alpha of 0.05 and a power of 0.80 of a two-tailed test, 64 participants will need to be included in each condition. The data will be analyzed according to the intent-to-treat principle. Multiple imputations will be used for missing observations at follow-ups. We intend to increase the sample size by 25% to compensate for dropout and potential loss of power due to clustering of data, resulting in 160 participants (80 in experimental condition and 80 in control condition).

### The program

‘Een Sprong Vooruit’ [A Jump Forward], which consists of 6 sessions, each 90 minutes long. In the first session, the participants will learn about emotions, anxiety, and depression and their experiences with their parent’s mental health problem. The adolescents learn about emotions they experience, and they will learn to recognize them. During this program, they will use a schedule to examine the relations among activating events, beliefs, emotional consequences and behavioral consequences. In the second session, the adolescents will learn about the relationship between activating events, beliefs, and emotional consequences. Beliefs can be optimistic or pessimistic and play a major role in the emotional consequences. The adolescents in the study will learn to recognize pessimistic beliefs. In the third session, adolescents will be taught several strategies to replace the pessimistic beliefs with optimistic beliefs. They will learn to recognize the (negative) pattern of their beliefs, replace them, and prove that the alternative belief is true. In the fourth session, adolescents will learn that in some situations, the beliefs cannot be replaced or changed and that they have to change their behavior to influence the emotional consequences. Therefore, the assignments will comprise both pleasant and less pleasant activities. Furthermore, they will learn to organize and structure large tasks. In the fifth session, the adolescents will learn about anxiety and the development of fear over time. By means of exposure, they will learn to divide fearful activities into small steps, practice those steps, and experience the decrease in anxiety. In the sixth and last session, the fearful tasks will be evaluated and practiced again. Furthermore, the adolescents will learn that social support sometimes makes things easier. They will practice asking people in their social environment for help. Finally, the adolescents will take a look at the future and talk about how they can influence it.

### Study outcome measures

For an elaborate overview of study outcome measures, see Table 1.

**Table 1:** Overview of assessments

	**Screening**	**T0**	**T1**	**T2**	**T3**	**T4**	**T5**
**Adolescent**							
Depression (CDI 2)	X	X	X	X	X	X	X
Anxiety (SCAS)	X	X	X	X	X	X	X
Suicidal ideation (CDI 2)	X	X	X	X	X	X	X
Response style (CRSQ)		X	X	X	X	X	X
Cognitive errors (CNCEQ-R)		X	X	X	X	X	X
Relational support (RSI)		X			X		X
**Parent**^**1**^							
Parental psychopathology (BSI)		X					
Parenting stress (OBVL)		X			X		X
Child depression (CDI 2 P)		X			X		X
Child anxiety (SCAS-P)		X			X		X

^1^Both parents will complete the questionnaires. Only parents who have absolutely no contact with children will be excluded.

### Screening measures

To assess the eligibility to participate, students will be screened for depressive and anxiety symptoms and suicidal ideation using the Children’s Depression Inventory 2 (CDI 2) and Spence Children Anxiety Scale (SCAS). Furthermore, students will be asked to complete some questions about parental psychopathology.

### Primary outcome measure

*Depression* in children and adolescents will be measured with the CDI 2 [47]. The CDI 2 contains 28 items, each consisting of three statements graded in severity from 0 to 2. This instrument has good internal consistency and convergent validity [48].

### Secondary outcome measures

*Anxiety* will be measured with the SCAS [49]. This 44-item self-report questionnaire rates the frequency of symptoms on a 4-point scale ranging from “never” to “always”. The scale has demonstrated high internal consistency, high concurrent validity, and adequate test-retest reliability across both child [50] and adolescent [51] samples.

*Suicidal ideation* will be measured with the suicide item of the CDI 2 [47], with scores of 0 (“no suicidal ideation”), 1 (“thoughts of wanting to kill oneself, with no intent to do so”), and 2 (“a desire to kill oneself, if given the chance”). Participants with a score of 2 will be excluded from the study and referred to a therapist within an institute of mental health care or a general practitioner.

*Response style* will be measured with the Children’s Response Style Questionnaire (CRSQ) [52]. This questionnaire consists of three subscales: ruminative response, distracting response, and problem solving. Items are rated on a 4-point scale ranging from “almost never” to “almost always”. The scale has moderate levels of internal consistency.

*Negative cognitive errors* will be measured with the Children’s Negative Cognitive Errors Questionnaire – Revised (CNCEQ-R) [53], which divides negative cognitive errors into five error categories, namely ‘overgeneralizing’ , ‘personalizing’ , ‘selective abstraction’ , ‘threat conclusion’ , and ‘underestimation of the ability to cope’. Items are rated on a 5-point scale from “not at all like I would think” to “almost exactly like I would think”. The questionnaire is a revised version of the CNCEQ, which has a good internal consistency and test-retest reliability [54].

*Parental emotional support* and *parental control* will be measured respectively with ‘Warmth versus Hostility’ and ‘Respect for Autonomy versus setting limits’ , which are two subscales of the Relational Support Inventory (RSI) [55]. Both subscales have six items rated on a 5-point scale ranging from “very untrue” to “very true”. The reliability varies from low to moderate.

*Parental psychopathology* will be measured with the Brief Symptom Inventory (BSI) [56]. The BSI is a short version (53 items) of the Symptom Checklist-90 (SCL-90) [57] divided into 9 symptom dimensions: Somatization, Obsessive-compulsive symptoms, Interpersonal sensitivity, Depression, Anxiety, Hostility, Phobic anxiety, Paranoid ideation, and Psychoticism. Items are rated on a 5-point scale from “not at all” to “extremely”. Both international [58] and national studies [59] showed that the questionnaire has good psychometric properties.

*Parenting stress* will be measured with the Opvoeding Belasting Vragenlijst (OBVL) [Parenting Stress Questionnaire] [60]. It is used to determine how parents experience their child, how they interact with their child, and how they feel about their own health. The questionnaire consists of 34 items scored on a 4-point scale ranging from “not true” to “very true”. This instrument has a good reliability and satisfactory validity [60].

*Depression of the child according to the parent* will be measured with the parent report of the CDI 2 [47]. Same as the child report, this questionnaire consists of 28 items, and for each item, there are three possible answers; 0 indicating the absence of symptoms, 1 indicating mild symptoms, and 2 indicating definite symptoms. The validity of this instrument is qualified as good [48].

*Anxiety of the child according to the parent* will be measured using the Spence Children’s Anxiety Scale-Parent Version (SCAS-P) [49]. The items of the SCAS-P were formulated as closely as possible to the corresponding items of the child version of the SCAS. The parent version of the SCAS demonstrates sound psychometric properties [61].

### Data analysis/statistical analysis

Data will be analyzed according to the intent-to-treat principle and will also be analyzed separately for the completers only. Multiple imputations will be used for missing observations at follow-ups. Regression analyses will be conducted to test differences in the development of depressive and anxiety symptoms between children in the intervention condition and children in the control condition. The results of the study will be reported in accordance with the CONSORT statement [45]. To investigate the mediating role of secondary outcome measures, such as response style and cognitive errors, mediation analyses will be performed in Mplus using bootstrap methods.

Possible baseline differences between the two conditions in demographic variables, depressive symptoms, and anxiety symptoms will be checked. Moreover, variables that show different distributions between the two groups will be entered as confounders in all models testing the effectiveness of the intervention.

## Discussion

This study protocol presents the design of a randomized controlled trial, which will evaluate the effect of a prevention program for depression and anxiety for adolescents with a high familial risk. The primary aim of the study will be to evaluate the effectiveness of the prevention program for depression and/or anxiety among 11-15 year old female adolescents. Our secondary aim will be to examine theoretically meaningful parent and child factors that possibly moderate or mediate the effect of the prevention program. It is hypothesized that adolescents in the intervention condition will show lower levels of depressive and anxious symptoms during the follow-up compared to adolescents in the control group without a prevention program. Furthermore, it is hypothesized that response style and cognitive errors will mediate the effect of the program and that parental psychopathology, parenting stress, parental emotional support, and parental control will moderate the effect of the prevention program.

### Strengths and limitations

The strength of the study is that it will conduct follow-up assessments at 12 months in addition to 6 months in both the intervention group and the control group. This will provide the opportunity to evaluate the long-term effects of this prevention program. Additional strength of the study is that in contrast to the most RCT studies, we will focus not only on the effectiveness of the program, but also on the mediators of change (i.e., how the intervention works). Finally, the prevention program will consist of only six sessions; therefore, we expect a low dropout rate.

This study also has limitations. We will not include a placebo intervention group, limiting the extent to which decreased depression and anxiety symptoms in the intervention group can be uniquely ascribed to the prevention program. Another study limitation is its selective design. It will enable a possible labeling or stigmatization effect, which can occur during the process of identification and participation of adolescents at risk in the prevention program.

### Implications for practice

Depression and anxiety are the most common internalizing disorders among adolescents. Prolonged and elevated levels of depression and anxiety can develop into clinical disorders. They can also cause significant distress and dysfunction, even at subclinical levels [8]. Given the prevalence of depression and anxiety and their high rates of recurrence, there is a need for effective assessment, treatment, and prevention. Schools can play an important role in the identification of children and adolescents with elevated depressive and anxiety symptoms. If the prevention program shows to be effective in reducing elevated levels of depression and anxiety and preventing adolescents from developing a clinical mental health disorder, our results will be relevant to practice. Hence, the intervention will be able to be used on large scale.

## Conclusion

This paper described the design of the study that will evaluate a prevention program for depression and anxiety in high-risk adolescents over a 12 months follow-up period. The targeted adolescents are at risk because both they and their parents have elevated levels of depression or anxiety. This study aims to contribute to the evidence-based prevention of depression and anxiety of adolescents.

## Competing interests

The authors declare that they have no competing interests.

## Authors’ contributions

SR is responsible for the data collection, data analysis, and for reporting the study results. All other authors are supervisors and grant applicators. All authors read and approved the final manuscript.

## Pre-publication history

The pre-publication history for this paper can be accessed here:

http://www.biomedcentral.com/1471-244X/13/316/prepub
